# The influence of anonymous peers on prosocial behavior

**DOI:** 10.1371/journal.pone.0185521

**Published:** 2017-10-09

**Authors:** Soowon Park, Jongho Shin

**Affiliations:** 1 Department of Education, Sejong University, Seoul, Republic of Korea; 2 Department of Education, Seoul National University, Seoul, Republic of Korea; Universidad de Alicante, ITALY

## Abstract

**Background:**

Peer influence on students’ maladaptive behaviors has been well documented; however, the influence on positive development is less acknowledged.

**Purpose:**

The purpose of this study was to examine anonymous peer influence on college students’ prosocial behavior, specifically behavior for the improvement of society (i.e., donating money or participating in social campaigns) via an experimental approach. The effects of indirect peer influence (IP) and direct peer influence (DP) on college students’ prosocial behavior were examined.

**Methods:**

A total of 125 college students participated in an online survey and laboratory experiment. Self-reported helping behavior, social concern goals, and empathy were measured by the online survey. In the laboratory experiments, reading of a prosocial paragraph (IP) and confederates’ prosocial behavior (DP) were manipulated. Participation in a signature campaign and money donation for illness were observed. Furthermore, 19 participants among those who donated were asked about their reasons for participating in such prosocial behavior.

**Results:**

Prosocial behavior of anonymous peers (confederates) exerts a profound influence on college students’ participation in a signature campaign and money donation, whereas the reading of a prosocial paragraph has no effect. Furthermore, no participants reported peer influence as a reason for engaging in prosocial behavior.

**Conclusion:**

This finding supports and extends recent research examining the positive impacts of anonymous peers on prosocial behavior. Prosocial behavior is not only a foundational and consistent aspect of personality, as previous studies report, but is also highly malleable and unstable in response to immediate situations.

## Introduction

One long-standing issue in the domain of psychological and educational research involves understanding why people contribute to charity or help someone they have never met [1,2]. Researchers treat internal characteristics, such as moral reasoning, empathy, or perspective taking, as crucial components of prosocial behavior, as such behavior is stable and consistent across situations [3,4]. Prosocial behavior, particularly behavior directed at improving society, has long been understood as an outcome of internal convictions or stable intrapersonal tendency and, thus, considered difficult to cultivate [5]. In line with these assumptions, previous longitudinal studies have found individual differences in prosocial behavior or that an individual’s prosocial disposition remains consistent in grades 4 to 12 [6], and between ages 4 to 24 [7], 6 to 12 [8], and 10 to 15 [9].

However, social psychologists have understood prosocial behavior in the light of social context and peer influence [10]. Evidence in social psychology has corroborated the notion that interpersonal situations can change human behavior in significant ways [11]. Empirical research has replicated peer influence on students’ social behavior or risk-taking [12–14]. Yet surprisingly little is known about how peers influence students’ positive social behavior, such as behavior for the improvement of society.

Social cognitive theory (SCT) [15] suggests two levels of analysis at which interpersonal interactions can affect prosocial behavior among humans: indirect peer influence (IP) and direct peer influence (DP) [16]. IP, which occurs at a level more distant than the immediate social environment, involves learning by observing the behavior of others via media, such as newspapers or television, which is also referred to as “symbolic modeling.” DP refers to the live modeling that occurs in one’s proximate and immediate social context, including via direct contact with another person, which is also referred to as “live modeling” [17]. Social cognitive theorists have documented that IP affects the behavior of humans [18]. The fictional models that appear on television, in newspapers, or in books have strong effects on behavior. One meta-analytic review reported that the social cognitive model can explain the increased risk of aggressive behaviors associated with playing violent video games [19]. These results support the notion that prosocial behavior may be improved by IP.

However, the influence of indirect exposure to the behavior of others on human behavior has been the subject of considerable controversy among researchers. Recent studies have found that IP (i.e., playing video games) has no effect on behavior [20]. Mere exposure to a violent model via media does not lead to behavioral change [21]. These controversial results are partially attributable to the fact that the effects of IP may be slight or may differ depending on the dependent variables, experimental procedures, or participants [16]. Therefore, the effect of IP should be further investigated in other domains, such as in fostering prosocial behavior. Previous studies have generally been conducted with children. Thus, the influence of indirect exposure on college students should also be examined.

DP is a major contributor to human behavior. Students change their behavior in response to their immediate social surroundings. One recent study, which found that moral decisions were affected by the judgments of others, reported that participants were more inclined to agree with a given action when confederates agreed with such an action in moral dilemma tasks [22]. Conformity [11] and the bystander effect [23] clearly demonstrate the influence of the response of others on the actions of individuals. Examination of the DP effect may even be more salient in collectivistic than in individual cultures, as people in the former are more likely to understand themselves as interdependent [24].

Many previous studies performed in adolescents and children found that peers are the most influential factor on students’ social adjustment and social functioning (For a detailed review, see [25]; Brechwald & Prinstein, 2011) [26]. Prosocial behavior of a friend is associated with 9th and 10th-grade adolescents’ prosocial goal pursuit and prosocial behavior [27]. Adolescents (mean age = 15.19) were more likely to engage in volunteering when their best friend and parents volunteered [28]. When children (mean age = 10) are involved in a prosocial group context, they are more likely to be accepted by peers and more likely to perceive themselves as socially competent, compared to an aggressive group context [29].

Natural field experiments also showed that social information has significant effects on donation. The amount of donated money depends on the number of bills and coins in the donation box [30]. Investigation of online fund-raising pages showed increases in previous donations increases future giving [31]. People are more likely to give larger donations when the donation request came from someone with whom they have social connections [32]. These field experiments support the notion that prosocial behavior (i.e., donation) is malleable in response to immediate situations.

However, to date, experimental approaches to reveal peer influence on prosocial behavior is very limited. Studies mentioned above used self-report or peer/teacher report survey designs. This may partly be attributed to the difficulties in arranging an ecologically valid way to measure prosocial behavior in a laboratory experiment. To the authors’ knowledge, only two recent studies have been performed using experimental approaches in the psychological field. These two studies utilized an online game [33] and chat room paradigm [34]. Adolescents were more likely to donate tokens to a peer group, rather than keep the token, when they were with unknown peers who provided prosocial feedback [33]. Intention to volunteer in community service is increased when a peer ostensibly endorses prosocial behavior in a chat room, especially if the peer has high social status [34]. These results suggest that anonymous peers contribute to increases in adolescents’ prosocial behavior. However, it is difficult to reflect practical reality entirely through online games and chat room settings, and the intention to become involved in prosocial behavior (i.e., volunteer community service) shown online does not guarantee actual prosocial behavior in the real world. Furthermore, the effect of peer influence on prosocial behavior, specifically behavior for the improvement of society, such as donating money or participating social campaigns, has not been demonstrated.

There have been more experimental approaches in the research field of economics. Reinstein & Riener (2012) found that the information regarding others’ (“leaders”) donations was influential to participants (“followers”), but only when their identities were revealed along with their donation amounts [35]. This experiment focused on the effects of reported identities of others when social connections between potential partners were already established. However, there is a possibility of not having social connections, which thus makes it hard to know the identity of others in natural settings.

In addition, experimental approaches in economics have studied peer effects on prosocial behavior using workers’ wage paradigm [36]. For instance, Gachter and co. utilized a three-person version of the bilateral gift exchange game, which consists of one “Employer” and two “Employees” [37] in an imaginary working situation. Even though previous studies revealed the crucial factor (e.g., payoff equality) to determine prosocial behavior among coworkers, these do not reflect daily situations and we cannot observe the effect of peers on prosocial behavior for the improvement of society.

## The current study

The current study administered a rigorous experimental design to examine the influence of anonymous peers on college students’ prosocial behavior, specifically with regards to donating money and participating in a signature campaign for a patient. Since the necessity for differentiation of the types of prosocial behavior has long been suggested [38], this study focused on prosocial behavior that is directly connected with the hope of contributing to the improvement of society.

The current study was designed to extend the understanding of peer influence on students’ prosocial behavior in several ways. First, anonymous peer influence was investigated. Based on the framework of SCT, prosocial behavior can be directly related to observing others within the context of social interactions and media influences. Here “others” can be a close friend, a teacher, parents, or someone that has never been met. In this study, the effect of anonymous peer influence was investigated because we encounter many strangers in the real world. If there is a strong anonymous peer influence on prosocial behavior, anonymous peers should be considered as critical factors for the development of interventions for enhancing prosocial behaviors and positive social adjustment. Even though there has been a considerable increase in theoretical and empirical studies exploring influencing factors on students’ prosocial behavior, such as social status [12, 39], few studies exist that examine the factors (e.g., anonymous peers) that could be utilized as effective interventions to promote students’ prosocial behavior. Second, the influence of anonymous peers was demonstrated via experimental approaches. Despite analytical advances in sampling and controlling for nuisance variables in correlational studies, experimental research still represents the most valid approach to warrant causal inferences and, thus, to investigate peer influence. Third, the present study focused on actual behavior in a “genuine” situation as a dependent variable. It has become increasingly important to transition from reliance on self-reports to reliance on observations of actual behavior [40]. However, previous studies typically measured participants’ antisocial behavior (i.e., aggressive behavior) by testing the students’ behavior of giving hot sauce to a partner who hates a strong spicy taste [41] or by giving shocks of various intensity towards an ostensible opponent [42]. Furthermore, prosocial behavior was measured by using an anonymous economic game [43], which measures how much the participants share the resources they have [44], by evaluating an individual’s propensity to cooperate in the Prisoner’s dilemma [45], or observing the extent to which they helped the experimenter [46,47]. Even though the results must be interpreted in light of the specific characteristics of the experimental procedure and conditions, the findings of the current study can be generalized to a real-world context because actual prosocial behavior in an ostensibly factual context was investigated. Fourth, this study focused on college students. Despite advances in the research understanding of prosocial behavior, studies involving participants beyond adolescence (i.e., college students) are still very limited. Since college is a critical period marking the beginning of adults’ integration into society, researchers should focus on the characteristics of prosocial behavior for the overall betterment of society in this period. Lastly, many previous studies related to peer influence on social behavior have been performed using western samples. However, the current study was conducted in a Korean population. Conducting such a study with non-western participants, such as Koreans, provides evidence and allows the broadening of theories, allowing for more cross-cultural generalization of results regarding peer influence on prosocial behavior.

In the current study, peer influences were divided into IP and DP. IP is defined as involving a distal interpersonal environment that affects behavior indirectly through messages, such as paragraphs read by participants. DP is defined as involving an immediate interpersonal surrounding, and it may include the presence of significant others. The effects of these two kinds of anonymous peer influence were experimentally tested. The central hypothesis guiding this study was that students’ prosocial behavior is under the influence of other’s behavior, even though they are not acquainted with the participants. Specifically, grounded in the literature on SCT and prosocial behavior, it was hypothesized that DP modeling would show an influence on fostering an individual’s prosocial behavior, whereas the effects of IP would be minimal. Furthermore, it was hypothesized that peer influence would be stronger over participants’ intrapersonal characteristics (i.e., self-reported helping behavior, social concern goals, empathy). In this study, prosocial behavior was operationalized as the participation in a signature campaign and money donations for those suffering from an illness.

## Methods

The current study was designed to investigate the causal relationship between peer effects (i.e., IP, DP) and prosocial behavior. To control the effects of intrapersonal characteristics (i.e., self-reported helping behavior, social concern goals, empathy), these were measured one week prior to the laboratory session through an online survey. In the laboratory session, peer effects were divided into two types of categories; IP and DP. To examine the effects of IP, whether reading written descriptions of people (i.e., university students) displaying prosocial behaviors affected the participants’ prosocial behavior or not was tested. To examine the effect of DP, whether direct exposure to confederates acting prosocially influences the participants’ prosocial behavior or not was tested. Participants’ prosocial behaviors were measured in three ways; (1) whether or not the participants contributed to a signature campaign for sick babies, (2) whether or not the participants donated money for sick babies, and (3) the amount of money donated to charity for sick babies.

### Participants and design

One hundred and fifty-two college students in Seoul, Republic of Korea, voluntarily participated in the study through the means of an online recruitment advertisement. All participants answered an online pre-questionnaire that was administered one-week prior to the laboratory experiment. However, 23 participants did not attend the laboratory experiment (14 did not show up and did not notify the researcher, and 9 canceled due to a sudden schedule change). Furthermore, four participants were excluded from data analysis because two reported suspicions about the authentic purpose of the study and the other two had personal connections with the researcher. This left 125 participants (71 female, 54 male, aged 18–34 years, *M*_age_ = 23.94, *SD*_age_ = 2.50) for the analysis. Demographic characteristics across conditions were presented in the Table 1. The majors were presented in the S1 Table. These classifications are followed those of Seoul National University (http://en.snu.ac.kr/undergraduate-programs).

**Table 1:** Demographic characteristics.

	Indirect Peer Influence						
	Absence		Presence				
	Direct Peer Influence		Direct Peer Influence				
	Absence (*n* = 30)	Presence (*n* = 34)	Absence (*n* = 31)	Presence (*n* = 30)	Total (*N* = 125)	*χ*^2^ or *F*	*p*
Age (*SD*)	23.47(2.08)	23.76(2.23)	24.29(2.76)	24.27(2.90)	23.94(2.50)	0.782	.506
Gender (M:F)	15:15	12:22	18:13	9:21	54:71	6.353	.093

*Note*. Standard deviations follow means in parentheses. M = male, F = female.

All participants were ethnically Korean and no measure for socioeconomic status was obtained. Participants were randomly assigned to one of four conditions; a 2 (absence vs. presence of IP) x 2 (absence vs. presence of DP) between-subjects design. Sample size was pre-determined using G*power 3.1 program [48], with results indicating a total of 92 participants was needed to reveal effects at a power level of .8, a medium effect size, and the significant α level of .05.

### Measures

Intrapersonal characteristics related to prosocial behavior (i.e., self-reported helping behavior, social concern goals, empathy), were measured during an online preliminary questionnaire session that took place one week prior to the laboratory session. In the laboratory session, participants’ prosocial behavior (i.e., contributing to a signature campaign, donating money, the amount of money donated to charity). Right after the laboratory session, three confederates answered questions about their level of prosocial behavior from a researcher.

#### Intrapersonal characteristics related to prosocial behavior

Self-reported helping behavior, social concern goals, and empathy were measured as intrapersonal characteristics related to prosocial behavior. All the scales were measured on a 5-point Likert scale (1 = *strongly disagree*, 3 = *neutral*, 5 = *strongly agree*), with higher scores denoting greater endorsement of the construct.

#### Self-reported helping behavior

In the online pre-questionnaire, individual’s helping behavior was measured through a degree of helping behavior measures developed and validated by Bar-Tal & Raviv (1979) [49]. Lee (1999) [50] validated the scale in Korean, and this scale was adopted and tailored to the purpose of the current study. The original scale includes items of helping behavior toward friends or relatives, but the purpose of the pre-questionnaire was to measure prosocial behavior towards strangers, so these items were omitted. Three items were chosen to measure an individual’s prosocial behavior toward strangers. Examples of these items include “I have helped a handicapped person or old person” and “I have helped a person in trouble, even though the person was a stranger who had no personal intimacy with me.” The Cronbach’s α of the scale was .67.

#### Social concern goals

In the online pre-questionnaire, social concern goals were measured because the prosocial behavior that is the focus of the current study stems from the value of social concern. The original scale was “social responsibility and concern goal” (McCollum, 2009) [51], and the Korean version was adopted from Shin and colleagues [52]. Four items were used, including “I consider it important to be considerate of others” and “I want to promote stability in the groups to which I belong.” The Cronbach’s α of the measure was .80.

#### Empathy

In the online pre-questionnaire, an individual’s empathy was measured through a degree of empathic concern in Davis (1983)’s [53] Interpersonal Reactivity Index (IRI). Jo and Lee (2010) [54] validated the scale in Korean, and four items were used in this study. The original scale included cons-trait items, but these items were excluded based on previous studies reporting a deficiency in using negative-keyed items [55,56]. Items of this scale included “I feel hurt when I see a poor old person” and “I feel pity and worry when I see a miserable person.” The Cronbach’s α of the measure was .77.

#### Assessments of prosocial behaviors

To measure students’ prosocial behaviors as dependent variables, the experimenter recorded whether each participant contributed to a signature campaign and the amount of money he or she donated to the pertinent charity. The experimenter explained that a charitable signature campaign was taking place to raise money for sick babies in the hospital who were unable to receive proper treatment due to a lack of money, and asked participants to join the signature campaign and make a donation. Detailed procedures are explained in the procedure section.

#### The amount of prosocial behavior requests across conditions

The appeal to the participants of the signature campaign and donation that the experimenter requested was measured. The confederates answered the online survey right after each experiment was done. One item (i.e., “Are the requests appealing?”) was developed and used to check the level of appeal across the conditions. The answer was measured via a 5-point Likert scale (1 = *strongly disagree*, 3 = *neutral*, 5 = *strongly agree*).

### Manipulation

#### Indirect peer influence (IP)

To manipulate the IP, three paragraphs were developed and employed in the pilot studies. The only difference between the control condition (i.e., absence of IP) and the experimental condition (i.e., presence of IP) was the type of paragraphs read by participants. Participants in the absence of IP conditions read the control paragraph that did not contain prosocial behavior but only neutral behavior such as traveling or attending a cooking class. On the other hand, participants in the presence of IP conditions read the paragraph containing prosocial behaviors. Paragraphs for the manipulation of IP were presented in the S1 and S2 Figs.

#### Pilot studies for developing materials for indirect peer influence conditions

Three paragraphs that were used for indirect peer influence conditions were developed through three pilot studies. Pilot studies were conducted with college students to test the validity of the IP condition manipulation. A total of 97 students (Mean age = 22.49, *SD* = 2.20) participated in the pilot studies. In the first study, 12 participants read five paragraphs, including three to five sentences that were designed to describe college students’ helping-related behaviors, including the words “help,” “donation,” and “voluntary service.” In addition, the paragraphs contained descriptions of positive consequences (i.e., receiving awards). Participants were told to write about the main theme of the paragraphs, the characteristics of the main characters, and the difficulties of the paragraphs. From the first pilot study, paragraphs required revision because participants tended to focus on the fact that the character was awarded. In three of the five paragraphs, all participants answered the main themes of the paragraphs as “receiving an award.” Therefore, the portrayal of the award was eliminated in the revised paragraphs. Instead, intrinsic rewards, such as feeling proud and happy, were involved. To enhance the sense of reality, detailed contents were added (e.g., community name, location in which the prosocial behavior occurred).

In the second pilot study, two different kinds of paragraphs were used: one was the modified version of the prior three paragraphs from the first pilot study and the other version served as a control paragraph which did not include prosocial-related words. A total of 62 participants were randomly assigned to read either the prosocial paragraph (*n* = 32) or the control paragraph (*n* = 30). In a similar manner to the first pilot study, participants were told to write about the characteristics of those who appeared in the paragraphs. According to the participants’ answers, the paragraphs were modified again. For the first paragraphs in particular, five participants (16%) described the protagonist’s characteristic as “showing off the company’s prosocial behavior” and nine participants (28%) answered “official.” Therefore, the contents about the company were deleted and all paragraphs were revised so that it read as if college students who performed prosocial behavior had written the paragraphs.

In the final pilot study, 23 participants read the final version of the three prosocial paragraphs (*n* = 12) and three control paragraphs (*n* = 11). These paragraphs were used in the main study. During subsequent questioning, all participants were requested to write about the possible ways of social contributions. In this preliminary statistical analysis, participants who read prosocial paragraphs reported more possible ways to contribute to society (*M* = 3.36, *SD* = 1.92) than those who read the control paragraphs (*M* = 1.27, *SD* = 1.19) in which the symbolic modeling of prosocial behavior was absent [*t*(21) = 3.10, *p* = .005, Cohen’s *d* = 1.31].

### Direct peer influence (DP)

To manipulate the DP condition, three student-confederates (always 1 male and 2 females) were employed. The number of confederates was pre-determined based on previous studies. Social influence varied depending on the number of people of a group [57]. Prior results suggest that three people lead to the most effective impact on the behavior of others [58]. Generally, the effects of DP were higher for groups of three people than groups of two; however, there was no significant difference in effect between groups of three and groups of four or five participants. Therefore, the ideal number of confederates was determined as three.

The only difference between the control conditions (i.e., absence of DP) and the experimental conditions (i.e., presence of DP) was the behaviors of confederates. The confederates in the absence of DP conditions did not behave prosocially. On the contrary, the confederates in the presence of DP conditions behaved prosocially. That is, in the absence of live DP, the groups of three confederates did not participate in the signature campaign or donate money but just left the laboratory. In contrast, the confederates subjected to DP conditions were willing to help and take part in the signature campaign and donation for sick babies. Since the confederates sitting near the door were the first to get their payment, and both the sheet for the signature campaign and donation box were near the door, participants could observe the behavior of other confederates. The setup of the laboratory session is presented in the S3 Fig.

### Asking the reason for participation in prosocial behavior

Among participants who donated a random sample were asked why they participated in prosocial behavior. The following question was asked through a phone call or in person: “What was your reason for donating money?”

### Procedures

The current study was performed from March to October, 2014. There were several practical issues for the reason why the experiments had been performed for several months. For each session, an average of three participants participated to allow for the manipulation of direct peer influence (i.e., participants should observe confederates’ actions). If the room is too big or there are too many participants, the researcher cannot ensure manipulation. The laboratory experiment was performed in the same place, which is actually a classroom, so we could only use the room when there was no class. In addition, it was not always easy to arrange a suitable time for three confederates, potential participations and one researcher. Furthermore, since the first semester started in March and ended in mid-June, and the second semester started in September, the experiment was adjourned during the summer vacation (i.e., from late-June to August) and the examination periods because voluntary participation was very low.

Participants completed a series of items during an online preliminary questionnaire session that took place one week prior to the laboratory session. Self-reported helping behavior, social concern goals, and empathy were measured. In order to make sure that participants did not recognize the true purpose of the study, two filler questionnaires, consisting of 24 items examining self-construals [59] and 23 items measuring free will beliefs [60], were presented in the questionnaire as well.

One week after the online questionnaire session, the participants attended a 20-minute laboratory experiment. Before the participants arrived for the experiment, three confederates (2 female, 1 male) were seated at the table in the experimental room near the door and they pretended to be waiting for the experiment to begin. After the actual participants arrived, the experimenter explained that the study was designed to examine thinking style and language processing. The setup of the laboratory session is presented in the S3 Fig. Since the average number of participants attending one session was three, a total of forty-two laboratory sessions conforming to one out of four experimental conditions were performed.

The participants spent 3 minutes reading the experimental paragraphs. IP manipulation was achieved by varying the paragraphs. Under the “absence of IP” condition, participants read paragraphs that did not describe prosocial behaviors. In contrast, under the “presence of IP” condition, participants read paragraphs that did describe prosocial behaviors, which were developed through the aforementioned pilot studies.

After they finished reading, participants performed a modified version of the scrambled sentence task [61]. In this task, sentences from the previous paragraph were placed in random order and participants were told to restore the sentences to their original order within 3 minutes. After the scrambled sentences task, participants completed a word decision task in which they received a list of 60 words and indicated whether each word had appeared in the previous paragraph.

Finally, the participants were informed that they had completed the experiment. The experimenter then informed them of the signature campaign and charity donation for sick babies who could not receive proper treatment due to a lack of money. The experimenter stressed that the participants were not required to participate in either. To ensure that the requests had equivalent appeal across the various conditions, the same instruction paragraph requesting the participants’ participation was used with all conditions; the experimenter memorized the paragraph and delivered it from memory. The paragraph requesting the participants’ participation is presented in the S4 Fig.

After all the instructions and tasks had been implemented, the participants were given 6,000 won (six 1,000-won bills; 1,000 won is equivalent to approximately 1 US dollar) for their participation in the study. Participants were given 1,000 won more than the amount originally indicated in the participation recruitment ad; i.e., participants expected to receive 5,000 won for their participation, but were actually given 6,000 won. This ploy ensured that all participants had sufficient money to donate to the charity as they received more than expected.

The DP condition involved manipulating the behavior of the three confederates. In the absence of the DP condition, the confederates neither participated in the signature campaign nor made a donation. In contrast, in the presence of DP condition, confederates took part in the signature campaign and made a donation. An average of three participants attended each session, and none of the participants in the same session knew each other. On average all three participants simultaneously decided whether to behave prosocially or not in the laboratory room. Participation in the signature campaign was not anonymous because participants had to sign their name in order to participate. Donations were anonymous but other participants were able to see who donated or not.

To assess the participants’ prosocial behaviors, the researcher recorded whether or not the participants took part in the signature campaign and the amount of money that each participant donated to the charity. Among participants who donated a random sample asked about the reason of participating in the prosocial behavior.

**Fig 1:**
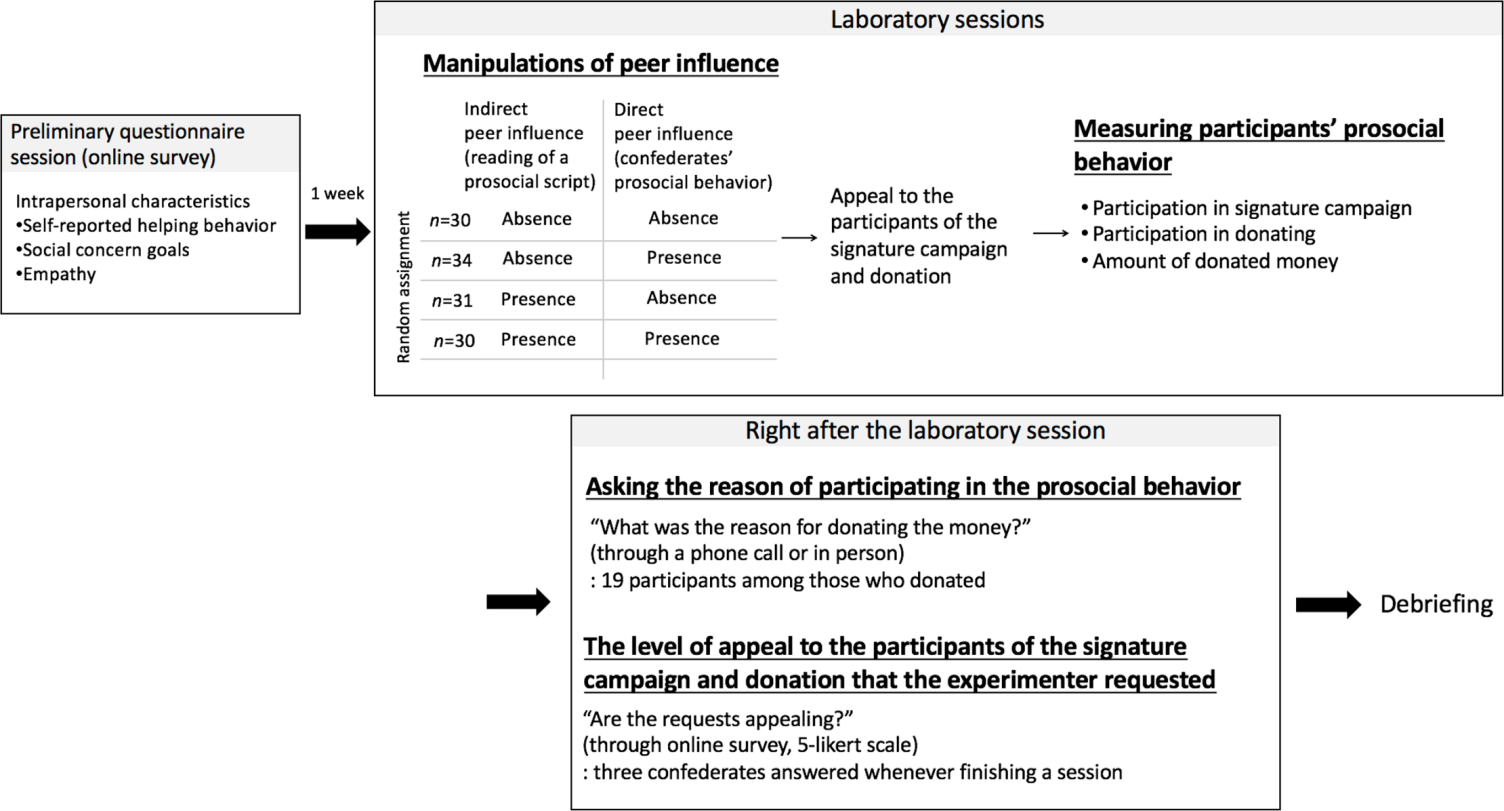
Diagram of the experimental procedure.

With regard to the manipulation check, all participants correctly answered the subsequent modified version of the scramble sentence task and word decision task, which means all participants read the paragraphs (manipulation check for indirect peer influence). To check for manipulation through DP, the experimenter observed whether or not the participants saw the actions of other confederates. When the observation was equivocal, the participants were asked about whether they saw the others’ (i.e., confederates’) behavior or not after finishing all the experiment. All participants reported that they saw what the other confederates did. Upon completion of the study, all participants were fully debriefed in person or by telephone, and again by e-mail. A diagram of the procedure is presented in Fig 1.

### Ethical standards

The Institutional Review Board of Seoul National University approved this study (IRB Number: 1408/002-003). All participants provided written informed consent to the experimenter before and after their participation in the study.

### Data analysis

#### Missing data analysis

The missing data proportion was absent across all items, except the value of social concern goals (count 3, 2.4%). A missing data proportion of less than approximately 5% is likely to be inconsequential for biases and loss of power, allowing for the use of list-wise deletion in regression analysis [62].

#### Statistical analysis

SPSS 18 (IBM, Somer, NY, USA) was used to analyze data. Descriptive statistics, including distribution of gender and means and standard deviations of age, are presented as demographic characteristics. To test homogeneity among the groups, the distribution of gender was compared using a *chi*-square test. Age, pre-questionnaire variables (i.e., helping behavior, social concern goals, empathy), and the level of request appeal were compared using a one-way analysis of variance (ANOVA) among the four groups.

Firstly, the differences in the proportion of participation in signature campaign and donation across the different conditions were examined through *chi*-square tests. The differences in the amount of money donated were examined through ANOVA. Multiple logistic regression was conducted to examine the difference of participation in the signature campaign. Three personal variables (i.e., helping behavior, social concern goals, empathy) as well as IP and DP conditions were entered as independent variables, and a dummy coded participation in the signature campaign (yes = 1, no = 0) was used as the dependent variable. To examine the difference of participation in donations multiple logistic regression was conducted. Independent variables were the same as the previous analysis (i.e., helping behavior, social concern goals, empathy, IP condition, DP condition) and a dummy coded participation in donation (yes = 1, no = 0) was used as a dependent variable. Lastly, multiple regression was performed to examine the effects of personal variables (i.e., helping behavior, social concern goals, empathy), IP, and DP on the amount of money donated. Variance Inflation Factors (VIFs were lower than 1.225) did not reveal any substantial multicollinearity problems [63].

The amount of money donated displayed a right skewed distribution (skewness = 3.38), a non-normal distribution (Kolmogorov-Smirnov *D* (115) = 0.44, *p* < .001), and unequal variance among groups (Levene’s *F* = 15.95, *p* < .001). Hence, log transformed values were used, but the result of significance was the same as the original value. Therefore, the results from the original values were reported for the convenience of understanding the coefficients. All statistical tests were two-tailed with the type 1 error set at 5%.

## Results

### Preliminary analyses for homogeneities of group characteristics

There were no differences in gender distribution (*χ*^2^ = 6.35, *p* > .05), age (*F*(3, 121) = 0.78, *p* > .05), helping behavior toward strangers (*F*(3, 121) = 1.21, *p* > .05), social concern goals (*F*(3, 121) = 1.18, *p* > .05), and/or empathy (*F*(3, 121) = 0.17, *p* > .05). Ratings of the confederates about the appeal of the experiment’s suggestions for participation in the signature campaign showed no differences (*F*(3, 25) = 0.15, *p* > .05). These results indicate homogeneities for group characteristics were verified.

### Anonymous peer influence on prosocial behavior

Table 2 presents the frequencies of participation in the signature campaign, frequencies of donations, and mean amount donated by each group. The frequencies of participation in the signature campaign and donation were larger when DP was present. The *chi*-square tests showed that there are significant relationships between participation and DP (*χ*^2^ *=* 34.891, *p* < .001 for the participation in the signature campaign, *χ*^2^ *=* 8.180, *p* = .005 for the participation on donation) and ANOVA showed the difference in amount money donated depending on DP (*F* = 12.715, *p* < .001), not IP (*F* = 0.248, *p* = .619).

**Table 2:** Descriptive statistics of dependent variables by conditions.

	Indirect Peer Influence						
	Absence		Presence				
	Direct Peer Influence		Direct Peer Influence				
	Absence (*n* = 30)	Presence (*n* = 34)	Absence (*n* = 31)	Presence (*n* = 30)	Total (*N* = 125)	*χ*^2^ or *F*	*p*
Frequencies of participation in signature campaign						Indirect:	
Nonparticipation	26	12	25	8	71	0.354	.591
Participation	4	22	6	22	54	Direct:	
						34.891	< .001
Frequencies of making donations						Indirect:	
Nonparticipation	29	22	26	20	97	0.329	.669
Participation	1	12	5	10	28	Direct:	
						8.180	.005
Amount of donated money^†^						Indirect:	
0 (did not donate)	29	22	26	20	97	0.248	.619
1,000	1	4	5	6	16	Direct:	
2,000	0	6	0	4	10	12.715	< .001
3,000	0	1	0	0	1		
6,000	0	1	0	0	1		
*M* (*SD*)	33.3(182.5)	735.3(1286.5)	161.3(373.88)	466.7(730.3)	360.0(827.0)		

Note.

^†^South Korean Won (KRW). 1000 KRW is approximately 1 United States dollar.

Table 3 reports the results of logistic (A. for predicting participation in the signature campaign, B. for predicting participation of donation) and multiple regression analyses (C. for predicting the amount of donated money). Self-reported helping behavior, social concern goals, empathy, IP influence, and DP were entered into the regression model as predictors of participating in the signature campaign. Only DP remained as a significant predictor (*b* = 2.51, *p* < .001). On the basis of the odds ratio, participants were 12.3 times more likely to participate in the signature campaign if the confederates took part in the campaign than when the confederates did not. Furthermore, only DP remained as a significant predictor (*b* = 1.28, *p* = .010) of donation. Participants were 3.6 times more likely to donate to charity if other confederates donated than when others did not donate. Multiple regression also showed the same result and that only DP predicted the amount of money donated (β = .29, *p* = .001). The results indicate a greater effect of DP in predicting participation in a signature campaign and donation than personal characteristics or IP.

**Table 3:** Logistic and multiple regression analysis predicting participation in the (A) signature campaign, (B) making donations, and (C) the amount of donated money.

Dependent Variable	(A) Participation in signature campaign					(B) Participation in donating					(C) Amount of donated money				
Predictor	*b*	*SE b*	Wald’s χ^2^	*p*	*e*^*b*^ (odds ratio)	*b*	*SE b*	Wald’s χ^2^	*p*	*e*^*b*^ (odds ratio)	*b*	SE *b*	beta	t	*p*
Constant	-4.176	2.239	3.478	.062	0.015	-1.958	2.166	0.817	0.366	0.141	18.085	679.634		.027	.979
Helping behavior	-0.070	0.398	0.030	.861	0.933	0.087	0.408	0.045	0.831	1.091	-27.436	132.948	-.020	-.206	.837
Social concern goal	0.686	0.396	2.995	.084	1.986	0.016	0.381	0.002	0.966	1.016	65.512	126.176	.051	.519	.605
Empathy	-0.039	0.485	0.007	.936	0.962	-0.171	0.495	0.120	0.730	0.843	-11.975	159.340	-.007	-.075	.940
Indirect peer influence	0.654	0.459	2.035	.154	1.924	0.440	0.465	0.897	0.344	1.553	-42.927	147.536	-.026	-.291	.772
Direct peer influence	2.511	0.471	28.430	< .001	12.316	1.280	0.495	6.693	0.010	3.598	483.813	147.103	.295	3.289	.001
Overall Model Fit															
-2 Log likelihood			127.76			120.742					Adjusted *R*^2^	.052			
Cox & Snell *R*^2^			.275			.065					*F*	2.320			
Nagelkerke *R*^2^			.369			.100					*p*	.048			

*Note*. *b* = unstandardized coefficient; beta = standardized coefficient

### Perceived reasons for prosocial behavior

Nineteen participants (68%) among those who donated (out of 28) were asked an open-ended question concerning the reason for participation in this prosocial behavior. Fourteen (74%) reported that they did so because it was “valuable.” Four (21%) reported emotional reasons, such as “feeling sorry for those suffering” or “having guilt for not helping poor people.” In addition, four (21%) answered that they donated money because they considered it an opportunity to help others. Two participants did not give any specific reasons, answering, “There was no specific reason.” There were no reports of social influence in the answers of the reason for engaging in prosocial behavior.

## Discussion

The present study investigated college students’ prosocial behavior, treating this behavior as a component of social interactions. Numerous naturalistic and observational studies about peer influence on human prosocial behavior have been performed since the 1970s, yet little research has been conducted with an experimental design–particularly on peer influence on prosocial behavior such as donating. This study helps to broaden the peer influence literature, particularly within a Korean sample.

Two theoretically plausible questions regarding social influences on prosocial behavior were tested. The first was whether a written description of an anonymous peer who displays prosocial behaviors can affect participants’ prosocial behavior. The other was whether direct exposure to actual anonymous peers (i.e. other confederates) acting prosocially can influence prosocial behavior. Indeed, college students’ immediate social context (i.e., actions of actual anonymous peers) proved to be a significant predictor of prosocial behavior. Even when taking into account personal characteristics, such as individual’s helping behavior, social concern goals, and empathy, the effects were incredibly influential.

The results show a significant importance of others in immediate surroundings over personal values. This result challenges traditional perspectives on prosocial behavior. Specifically, that internal norms and convictions are typically believed to be the strongest predictors of prosocial behavior, such as charitable donation [5], with the effect of the influence of others remaining largely unacknowledged. Thus, a sole focus on intrapersonal characteristics may fail to fully understand of the prosocial behavior in college students.

Only DP exerted a strong influence on college students’ prosocial behavior. The results clearly showed the effect of conformity on prosocial behaviors, even when the individual had no personal relationship with the other present. Conformity is one of the most important social influences on general human behavior [58]. People overtly change their behavior in response to majority behaviors. A meta-analysis indicated that participants from collectivistic countries, such as Korea, showed a higher level of conformity than did those from individualistic countries [64]. One important underlying motivation for conforming to the behavior of others is the need for social approval. The central mechanism of conformity is normative influence. Normative influence is defined as social influence that arises from the need to be accepted by others [65]. That is, an individual does not wish to be perceived as deviating from group behavior [66,67]. Because making an effort to help a person in trouble and donating money to the poor are generally regarded positively across cultures [2], normative influence is likely to be especially strong in the domain of prosocial behavior. Prosocial behaviors are dubbed as “good” and “nice.” When other people (three confederates in the present study) engage in prosocial behaviors, an individual tends to behave similarly to obtain social approval by meeting the social norm.

The social norm approach offers a possible mechanism for enhancing students’ prosocial behavior [68]. Many adolescents have exaggerated perceptions of others’ behaviors. For instance, an adolescent who is a heavy drinker of alcohol may believe that he or she drinks less than the majority [69]. In the case of prosocial behaviors, making students aware of social norms in which many society members work for the betterment of society may enhance their own prosocial behavior. These explanations and mechanisms warrant further examination in future studies.

Reading paragraphs about another’s prosocial behaviors did not affect participants’ prosocial behaviors (i.e., participating in the signature campaign or donating money). Many previous studies have posited that reading paragraphs or being exposed to stimuli associated with a certain topic can induce motivational, emotional, cognitive, and behavioral changes [61,70–73]. According to Bandura, news and entertainment media can encourage or discourage moral behavior [74].

However, in the present study, there was little influence of IP when this condition entailed reading prosocial paragraphs. Since there are few experimental studies revealing indirect influence on prosocial behavior, specifically a “high” level of prosocial behavior such as making donations, it is hard to conclude the reasons underlying this result. There could be four possible reasons for why IP has little effect on prosocial behavior. First, reading three short paragraphs may not be sufficient for finding the effect of IP. Reading paragraphs is considered as an appropriate way to teach desirable behaviors in an educational setting. For instance, educators and parents attempt to make students learn desirable behavior through the means of reading the biography of a great person. However, reading is considered a relatively vague and indirect way to transfer information about other peoples behaviors than watching videos [75] or pictures [76]. According to the dual-coding approach, images or movies, rather than words, are more powerful in activating cognitive processes that could affect an individual’s behavior [77]. Therefore, it is still possible that other ways of IP manipulation could enhance students’ prosocial behavior.

Second, reading paragraphs a single time may not be sufficient to induce the effects of symbolic modeling. Previous research suggests that repeated exposure to aggressive stimuli is necessary for inducing long-lasting changes of students’ behavior [78,79]. Since the participants of this study only read short paragraphs a single time, it might not be sufficient for the symbolic modeling to have an impact on fostering prosocial behavior. Repeated exposures to prosocial paragraphs could foster an individual’s prosocial behavior.

The third possible reason for not being able to find the effects of IP is the way prosocial behavior is measured. Previous results suggest that even a subtle change of administration methods for measuring prosocial behavior could be more powerful than the presence of IP (i.e., conducting different kinds of video games) [20]. Tear and Nielsen (2013) [20] used the pen-drop method to measure participants’ prosocial behavior. Frequencies of helping pick up pens were counted to quantify the extent of prosocial behavior. Contrary to expectations, there was no difference depending on what kind of game the participants saw. Rather, the most important aspect was the timing of when the researcher dropped pens (i.e., after finishing the manipulation or in the middle of the experiment). These previous results showed that the specific method used to measure prosocial behavior could be a key factor for finding the effects of IP. Few studies have used designs to examine whether IP could lead to changes in real prosocial behavior for others the individual has never met. In the present study, participation in the signature campaign and charity for sick babies were used as the dependent variables. Spending time and money for enhancing the quality of life of someone the individual has never met is considered to be a characteristic that is hard to develop [80]. Performing this type of prosocial behavior is much harder than other prosocial behaviors, such as cooperation or helping close friends. Therefore, just reading a paragraph may not be sufficient for inducing prosocial behavior for society at all.

Observational learning occurs differently in various fields (Bandura et al., 1969). The results of this study address the weakness of SCT, namely its overly broad approach in explaining social phenomena [81]. By narrowing the concept of modeling, the precise effects of other’s influence can be investigated. The present study was the first to investigate the role of IP by examining the effect of reading a prosocial paragraph on actual prosocial behaviors. More studies with multiple measures and valid stimuli need to be conducted to replicate and confirm these findings.

Whether violent games can increase aggression among students [19] or diminish prosocial behavior [20] has been a controversial issue among educators, researchers, and policy makers. Our results showed that, in a specific context where the targeted prosocial behavior involved alleviating the suffering of a fellow member of society, the effect of IP, as represented by reading paragraphs, was faint. Instead, giving students the opportunity to be involved in a group whose members behave prosocially could be a more effective way to enhance their prosocial behavior.

Interestingly, there was no report of social influence in the answers of the reason for engaging in prosocial behavior. Participants did not perceive the effect of others’ behavior on their own. Participants answered that they performed the behavior of their own volition. Previous research has continuously revealed that people are not aware of the fact that contextual factors influence their behavior (e.g., Jonas et al., 2002; Shariff & Norenzayan, 2007). Since there is seemingly no explicit external cause for producing certain behaviors, people usually think that they know why they start the behavior or what triggers it.

There is a critical advantage of the perception that prosocial behavior is rooted in inner desires. Having experiences of performing prosocial behavior helps to foster the self-concept of prosocial being [82]. Previous studies found that engaging with volunteer activity would develop one’s civic identity and political socialization process, and would instill social responsibility [83,84]. This effect may be maximized when individuals think that they are performing the behavior because they want to. If people think that there is a mandatory reason for engaging in prosocial behavior, the intention to perform the behavior declines in the future [85].

Perceived autonomy has a strong relationship with prosocial behavior engagement in college students and volunteer workers [86]. The feeling of autonomy is one of the most important factors that motivate a student to engage in activities [87]. Furthermore, the extent to which prosocial behavior is autonomous predicts the helper’s psychological well-being [88]. Therefore, it is crucial to develop the environment in order to provide an opportunity to behave prosocially with a feeling of control. The present study suggests that the presence of live modeling is one possible answer.

Finally, it is intriguing to note that participants’ intrapersonal characteristics (i.e., self-reported helping behavior, social concern goals, empathy) had no relationships with prosocial behavior when controlling IP and DP. In further analysis (results were not shown), spearman’s rho showed that participation in the signature campaign was positively correlated with self-reported helping behavior (*r* = .473, *p* = .008) and a social concern goal (*r* = .401, *p* = .028) in the control group (*n* = 30). These results showed that self-reported helping behavior and a social concern goal can be related with prosocial behavior when there is no manipulation of peer influence. Especially, the intrapersonal characteristics (i.e., self-reported helping behavior, social concern goal) can predict a “low” level of prosocial behavior such as participation in a signature campaign, which does not cause personal loss. Our finding about the crucial influence of DP over the intrapersonal characteristics is important for designing educational programs or policies aimed at encouraging prosocial behavior, specifically “high” level prosocial behavior such as making donations.

The findings of the current study raise further questions for future research. First, this study only showed a benefit of the presence of DP, relative to an absence of DP. It has not been demonstrated that whether presence of DP improves participants’ prosocial behavior, whether absence of DP undermines their prosocial behavior, or both. Further study should be performed to reveal these unanswered questions. Second, further study is needed to elucidate moderating factors of the modeling procedure. This study did not examine possible moderators of modeling effects, and numerous factors may enhance or attenuate DP effects. For instance, characteristics of confederates may moderate the effect of social influence. According to Bandura, the modeling effect was increased when the model was similar to the participants [89], with greater degrees of perceived similarity increasing the likelihood that the participant would imitate the model. Gender, age, appearance, group membership, or even a minimal social bond (e.g., having the same birthdate) are possible moderators of the impact of others’ behaviors [90,91]. Third, another important question that remains is the duration of the DP effect on an individual’s behavior. In the present study, the effects of confederates’ behaviors were examined by measuring prosocial behaviors immediately after the manipulation. However, it cannot be guaranteed that participants who exhibited prosocial behaviors in the laboratory would continue to engage in such behaviors in their everyday lives. It is possible that the effects were short lived. Thus, additional research is needed to elucidate the temporal nature of the effects of direct social influence on prosocial behavior. Fourth, it would be valuable to determine the consequences of prosocial behaviors. Even though the participants did not realize that they were influenced by the behavior of others, it is possible that their prosocial behavior led to improvements in their well-being or self-esteem [92,93]. A greater understanding of these issues would give educators clear reasons for creating social environments that encourage individuals to engage in prosocial behavior.

### Conclusion

Current findings highlight the importance of anonymous peer influence on college students’ prosocial behavior toward the betterment of society, even after critical intrapersonal factors are taken into account. Findings suggest that prosocial behavior of anonymous peers may be very helpful in fostering students’ prosocial behavior (a more than 12-fold increase in participation of a social campaign and 3-fold increase in donation). Findings also highlight the importance of the immediate social context as one of the possible ways to encouraging college students’ prosocial behaviors with feelings of autonomy. Prosocial behavior that is directed at improving society is both a stable trait that is consistent across situations and unstable in response to immediate social situations. Therefore, educators and researchers should consider the effect of direct social context when understanding students’ prosocial behavior when developing educational programs or establishing policies to increase college students’ prosocial behavior.

## Supporting information

S1 Table (DOCX)

S1 Fig (DOCX)

S2 Fig (DOCX)

S3 Fig (DOCX)

S4 Fig (DOCX)

## References

[1] Berndt TJ. Effects of Friendship on Prosocial Intentions and Behavior. Child Dev. 2014;52: 636–643.

[2] Davidio J, Piliavin JA, Schroeder D, Penner L. The social psychology of prosocial behavior. New York: Psychology Press; 2006.

[3] Calhoun C. Setting the moral compass: Essays by women philosophers [Internet]. New York: Oxford University Press; 2004 Available: http://books.google.com/books?hl=en&lr=&id=SY6mgPFzkkYC&oi=fnd&pg=PR9&dq=Setting+the+Moral+Compass:+Essays+by+wonmen+philosophers&ots=x7kQOnumsf&sig=Roz_Fi1vSSBgPmaW3U8eUJ9nj0k

[4] Kant I, Mary G. Kant: the metaphysics of morals. Cambridge University Press; 1996.

[5] van der Linden S. The helper’s high: Why it feels so good to give. Ode Mag. 2011;8: 26–27.

[6] Flynn E, Ehrenreich SE, Beron KJ, Underwood MK. Prosocial Behavior: Long-term Trajectories and Psychosocial Outcomes. Soc Dev. 2015;24: 462–482. doi: 10.1111/sode.12100 26236108 10.1111/sode.12100 PMC4517683

[7] Eisenberg N, Guthrie IK, Murphy BC, Shepard S a, Cumberland A, Carlo G. Consistency and development of prosocial dispositions: a longitudinal study. Child Dev. 1999;70: 1360–1372. doi: 10.1111/1467-8624.00100 10621961 10.1111/1467-8624.00100

[8] Cote S, Tremblay RE, Nagin D, Zoccolillo M, Vitaro F. The development of impulsivity, fearfulness, and helpfulness during childhood: patterns of consistency and change in the trajectories of boys and girls. J Child Psychol Psychiatry. 2002;43: 609–618. doi: 10.1111/1469-7610.00050 12120857 10.1111/1469-7610.00050

[9] Nantel-Vivier A, Kokko K, Caprara GV, Pastorelli C, Gerbino MG, Paciello M, et al Prosocial development from childhood to adolescence: A multi-informant perspective with Canadian and Italian longitudinal studies. J Child Psychol Psychiatry. 2009;50: 590–598. doi: 10.1111/j.1469-7610.2008.02039.x 19207631 10.1111/j.1469-7610.2008.02039.x

[10] Fiske S, Harris L, Cuddy A. Why ordinaly people torture enemy prisoners. Science (80-). 2004;306: 1482–1483. Available: http://psychoanalystsopposewar.org/resources_files/Why_Ordinarty_People_Torture_Enemy_Prisoners_Fiske.pdf 10.1126/science.1103788 15567841

[11] Cialdini R, Goldstein N. Social influence: compliance and conformity. Annu Rev Psychol. 2004;55: 591–621. doi: 10.1146/annurev.psych.55.090902.142015 14744228 10.1146/annurev.psych.55.090902.142015

[12] Berger C, Rodkin PC. Group Influences on Individual Aggression and Prosociality: Early Adolescents Who Change Peer Affiliations. Soc Dev. 2012;21: 396–413. doi: 10.1111/j.1467-9507.2011.00628.x

[13] Weigard A, Chein J, Albert D, Smith A, Steinberg L. Effects of anonymous peer observation on adolescents’ preference for immediate rewards. Dev Sci. 2014;17: 71–78. doi: 10.1111/desc.12099 24341973 10.1111/desc.12099 PMC3869036

[14] Smith AR, Chein J, Steinberg L. Peers increase adolescent risk taking even when the probabilities of negative outcomes are known. Dev Psychol. 2014;50: 1564–1568. doi: 10.1037/a0035696 24447118 10.1037/a0035696 PMC4305434

[15] Bandura A. Social cognitive theory: an agentic perspective. Annu Rev Psychol. 2001;52: 1–26. doi: 10.1146/annurev.psych.52.1.1 11148297 10.1146/annurev.psych.52.1.1

[16] Bandura A, Blanchard EB, Ritter B. Relative efficacy of desensitization and modeling approaches for inducing behavioral, affective, and attitudinal changes. J Pers Soc Psychol. 1969;13: 173–99. Available: http://www.ncbi.nlm.nih.gov/pubmed/5389394 5389394 10.1037/h0028276

[17] Charlop-Christy MH, Le L, Freeman KA. A Comparison of Video Modeling with In Vivo Modeling for Teaching Children with Autism. J Autism Dev Disord. 2000;30: 537–552. 11261466 10.1023/a:1005635326276

[18] Liebert R, Sprafkin J. The early window: The effect of television on children and youth. New York: Pergamon; 1988.

[19] Anderson C, Shibuya A, Ihori N, Swing E, Bushman B, Sakamoto A, et al Violent video game effects on aggression, empathy, and prosocial behavior in eastern and western countries: a meta-analytic review. Psychol Bull. 2010;136: 151–173. doi: 10.1037/a0018251 20192553 10.1037/a0018251

[20] Tear MJ, Nielsen M. Failure to demonstrate that playing violent video games diminishes prosocial behavior. PLoS One. 2013;8: e68382 doi: 10.1371/journal.pone.0068382 23844191 10.1371/journal.pone.0068382 PMC3700923

[21] Elson M, Ferguson CJ. Does Doing Media Violence Research Make One Aggressive? Eur Psychol. 2014;19: 68–75. doi: 10.1027/1016-9040/a000185

[22] Kundu P, Cummins DD. Morality and conformity: The Asch paradigm applied to moral decisions. Soc Influ. 2013;8: 268–279. doi: 10.1080/15534510.2012.727767

[23] Checkroun P, Markus B. The bystander effect and social control behavior: the effect of the presence of others on people’s reactions to norm violations. Eur J Soc Psychol. 2002;32: 853–867. Available: http://onlinelibrary.wiley.com/doi/10.1002/ejsp.126/abstract

[24] Markus HR, Kitayama S. Culture and the self: Implications for cognition, emotion, and motivation. Psychol Rev. American Psychological Association; 1991;98: 224–253. doi: 10.1037//0033-295X.98.2.224

[25] Brechwald WA, Prinstein MJ. Beyond homophily: A decade of advances in understanding peer influence processes. J Res Adolesc. 2011;21: 166–179. doi: 10.1111/j.1532-7795.2010.00721.x 23730122 10.1111/j.1532-7795.2010.00721.x PMC3666937

[26] Prinstein MJ, Brechwald W, Cohen GL. Susceptibility to peer influence: Using a performance-based measure to indentify adolescent males at heightened risk for deviant peer socializtion. Dev Psychol. 2011;47: 1167–1172. doi: 10.1037/a0023274 21463036 10.1037/a0023274 PMC3348704

[27] Barry CM, Wentzel KR. Friend influence on prosocial behavior: The role of motivational factors and friendship characteristics. Dev Psychol. 2006;42: 153–163. doi: 10.1037/0012-1649.42.1.15 16420125 10.1037/0012-1649.42.1.15

[28] Van Goethem AAJ, van Hoof A, van Aken MAG, Orobio de Castro B, Raaijmakers QAW. Socialising adolescent volunteering: How important are parents and friends? Age dependent effects of parents and friends on adolescents’ volunteering behaviours. J Appl Dev Psychol. Elsevier Inc.; 2014;35: 94–101. doi: 10.1016/j.appdev.2013.12.003

[29] Chung-Hall J, Chen X. Aggressive and prosocial peer group functioning: Effects on children’s social, school, and psychological adjustment. Soc Dev. 2010;19: 659–680. doi: 10.1111/j.1467-9507.2009.00556.x

[30] Martin R, Randal J. How is donation behaviour affected by the donations of others? J Econ Behav Organ. 2008;67: 228–238.

[31] Smith S, Windmeijer F, Wright E. Peer effects in charitable giving: Evidence from the (Running) field. Econ J. 2013;125: 1053–1071. doi: 10.1111/ecoj.12114

[32] Meer J. Brother, can you spare a dime? Peer pressure in charitable solicitation. J Public Econ. Elsevier B.V.; 2011;95: 926–941. doi: 10.1016/j.jpubeco.2010.11.026

[33] van Hoorn J, van Dijk E, Meuwese R, Rieffe C, Crone E a. Peer Influence on Prosocial Behavior in Adolescence. J Res Adolesc. 2014;26: n/a-n/a. doi: 10.1111/jora.12173

[34] Choukas-Bradley S, Giletta M, Cohen GL, Prinstein MJ. Peer Influence, Peer Status, and Prosocial Behavior: An Experimental Investigation of Peer Socialization of Adolescents’ Intentions to Volunteer. J Youth Adolesc. Springer US; 2015;44: 2197–2210. doi: 10.1007/s10964-015-0373-2 26525387 10.1007/s10964-015-0373-2 PMC5985442

[35] Reinstein D, Reinstein D, Riener G. Reputation and Influence in Charitable Giving : An Experiment. Theory Decis. 2012;72: 221–243.

[36] Thöni C, Gächter S. Peer effects and social preferences in voluntary cooperation: A theoretical and experimental analysis. J Econ Psychol. 2015;48: 72–88. doi: 10.1016/j.joep.2015.03.001

[37] Gächter S, Nosenzo D, Sefton M. Peer effects in pro-social behavior: Social norms or social preferences? J Eur Econ Assoc. 2013;11: 548–573. doi: 10.1111/jeea.12015 28553193 10.1111/jeea.12015 PMC5443401

[38] Eisenberg N, Fabes R, Spinrad T. Prosocial development In: Damon W, Lerner RM, editors. Handbook of Child Psychology. Hoboken, NJ, USA: John Wiley & Sons, Inc.; 2007 doi: 10.1002/9780470147658

[39] Rodkin PC, Ryan AM, Jamison R, Wilson T. Social Goals, Social Behavior, and Social Status in Middle Childhood. Dev Psychol. 2012;49: 1139–1150. doi: 10.1037/a0029389 22822934 10.1037/a0029389

[40] Baumeister RF, Vohs KD, Funder DC. Psychology as the science of self-reports and finger movements. Perspect Psychol Sci. 2007;2: 396–403. doi: 10.1111/j.1745-6916.2007.00051.x 26151975 10.1111/j.1745-6916.2007.00051.x

[41] Lieberman J, Solomon S, Greenberg J, McGregor H. A hot new way to measure aggression: Hot sauce allocation. Aggress Behav. 1999;25: 331–348. Available: http://onlinelibrary.wiley.com/doi/10.1002/(SICI)1098-2337(1999)25:5%3C331::AID-AB2%3E3.0.CO;2-1/abstract

[42] Giancola PR, Parrott DJ. Further evidence for the validity of the Taylor Aggression Paradigm. Aggress Behav. 2008;34: 214–229. doi: 10.1002/ab.20235 17894385 10.1002/ab.20235

[43] Matsumoto Y, Yamagishi T, Li Y, Kiyonari T. Prosocial behavior increases with age across five economic games. PLoS One. 2016;11: 1–16. doi: 10.1371/journal.pone.0158671 27414803 10.1371/journal.pone.0158671 PMC4945042

[44] Shariff AF, Norenzayan A. God is watching you: priming God concepts increases prosocial behavior in an anonymous economic game. Psychol Sci. 2007;18: 803–809. doi: 10.1111/j.1467-9280.2007.01983.x 17760777 10.1111/j.1467-9280.2007.01983.x

[45] Mengel F. Computer games and prosocial behaviour. PLoS One. 2014;9: 1–5. doi: 10.1371/journal.pone.0094099 24718635 10.1371/journal.pone.0094099 PMC3981774

[46] Baron RA. The Sweet Smell of… Helping: Effects of Pleasant Ambient Fragrance on Prosocial Behavior in Shopping Malls. Personal Soc Psychol Bull. 1997;23: 498–503. doi: 10.1177/0146167297235005

[47] Van baaren RB, Holland RW, Kawakami K, Knippenberg A Van. Mimicry and Prosocial Behavior. Psychol Sci. 2004;15: 71–74. doi: 10.1111/j.0963-7214.2004.01501012.x 14717835 10.1111/j.0963-7214.2004.01501012.x

[48] Faul F, Erdfelder E, Lang A-G, Buchner A. G*Power 3: a flexible statistical power analysis program for the social, behavioral, and biomedical sciences. Behav Res Methods. 2007;39: 175–191. Available: http://www.ncbi.nlm.nih.gov/pubmed/17695343 17695343 10.3758/bf03193146

[49] Bar-Tal D., & Raviv A. (1979). Consistency of helping-behavior measures. Child Development, 50(4), 1235–1238.

[50] Lee, H.-S. (1999). A study of the volunteering service experiences of the youth on altruism (Master’s thesis). Myong Ji University.

[51] McCollum DL. What Are the Social Values of College Students?: A Social Goals Approach. J Coll Character. 2009;6: Published Online.

[52] Shin J., Jin S., Rhee S. H., Park S., Kim Y.-E., & Kim S.-H. (2014). A study on the difference in goal contents and goal-seeking processes according to expertise levels of professional engineers and artists. The Korean Journal of Educational Psychology, 28(3), 455–476.

[53] Davis M. (1983). Measuring individual differences in empathy: Evidence for a multidimensional approach. Journal of Personality & Social Psychology, 44(1), 113–126.

[54] Jo H., & Lee M. (2010). The mediating effect of prosocial behavior in the relation between empathic ability and psychological well-being. Korean Journal of Youth Studies, 17(11), 140–159.

[55] Barnette JJ. Effects of Stem and Likert Response Option Reversals on Survey Internal Consistency: If You Feel the Need, There is a Better Alternative to Using those Negatively Worded Stems. Educ Psychol Meas. 2000;60: 361–370. doi: 10.1177/00131640021970592

[56] Schriesheim CA, Eisenbach RJ, Hill KD. The Effect of Negation and Polar Opposite Item Reversals on Questionnaire Reliability and Validity: An Experimental Investigation. Educ Psychol Meas. 1991;51: 67–78. doi: 10.1177/0013164491511005

[57] Milgram S, Bickman L, Berkowitz L. Note on the drawing power of crowds of different size. J Pers Soc Psychol. 1969;13: 79–82. doi: 10.1037/h0028070

[58] Asch SE. Opinions and Social Pressure. Sci Am. 1955;193: 31–35. doi: 10.1038/scientificamerican1155-31

[59] Singelis TM. The Measurement of Independent and Interdependent Self-Construals. Personal Soc Psychol Bull. 1994;20: 580–591. doi: 10.1177/0146167294205014

[60] Paulhus DL, Carey JM. The FAD-Plus: measuring lay beliefs regarding free will and related constructs. J Pers Assess. 2011;93: 96–104. doi: 10.1080/00223891.2010.528483 21184335 10.1080/00223891.2010.528483

[61] Bargh J, Gollwitzer P, Lee-Chai A, Barndollar K, Trötschel R. The automated will: nonconscious activation and pursuit of behavioral goals. J Pers Soc Psychol. 2001;81: 1014–1027. Available: http://www.pubmedcentral.nih.gov/articlerender.fcgi?artid=3005626&tool=pmcentrez&rendertype=abstract 11761304 PMC3005626

[62] Graham JW. Missing data analysis: Making it work in the real world. Annu Rev Psychol Psychol. 2009;60: 549–576. doi: 10.1146/annurev.psych.58.110405.085530 18652544 10.1146/annurev.psych.58.110405.085530

[63] Neter J, Kutner M, Wasserman W. Applied linear statistical models: Regression, analysis of variance, and experimental designs. Chicago: Irwin; 1990.

[64] Bond R, Smith PB. Culture and Conformit: A Meta-Analysis of Studies Using Asch ‘ s (1952b, 1956) Line Judgment Task. Psychol Bull. 1996;119: 111–137.

[65] Deutsch M, Gerard HB. A study of normative and informational social influences upon individual judgment. J Abnorm Soc Psychol. 1955;51: 629–636. doi: 10.1037/h0046408 10.1037/h0046408 13286010

[66] Wood W, Lundgren S, Oueleette J, Busceme S, Blackstone T. Minority influence: a meta-analytic review of social influence processes. Psychol Bull. 1994;115: 323–345. Available: http://psycnet.apa.org/journals/bul/115/3/323/ 8016284 10.1037/0033-2909.115.3.323

[67] Yanovitzky I, Rimal RN. Communication and Normative Influence: An Introduction to the Special Issue. Commun Theory. 2006;16: 1–6. doi: 10.1111/j.1468-2885.2006.00002.x

[68] Perkins W. The emergence and evolution of the social norms approach to substance abuse prevention The social norms approach to preventing school and college age substance abuse: A handbook for educators, counselors, and clinicians. San Francisco, CA: Jossey-Bass; 2003 pp. 3–17.

[69] Segrist DJ, Corcoran KJ, Jordan-Fleming MK, Rose P. Yeah, I Drink… but Not as Much as Other Guys: The Majority Fallacy among Male Adolescents. N Am J Psychol. 2007;9: 307–320.

[70] Bargh J, Chartrand T. The unbearable automaticity of being. Am Psychol. 1999;54: 462–479. Available: http://psycnet.apa.org/journals/amp/54/7/462/

[71] Fitzsimons G, Bargh J. Thinking of you: nonconscious pursuit of interpersonal goals associated with relationship partners. J Pers Soc Psychol. 2003;84: 148–164. Available: http://psycnet.apa.org/journals/psp/84/1/148/ 12518976 PMC3011819

[72] Oettingen G, Grant H, Smith P. Nonconscious goal pursuit: Acting in an explanatory vacuum. J Exp Soc Psychol. 2006;42: 668–675. Available: http://www.sciencedirect.com/science/article/pii/S0022103105001216

[73] Shidlovski D, Hassin RR. When pooping babies become more appealing: the effects of nonconscious goal pursuit on experienced emotions. Psychol Sci. 2011;22: 1381–1385. doi: 10.1177/0956797611417135 21987695 10.1177/0956797611417135

[74] Bandura A. Social foundations of thought and action: A social cognitive theory Frontiers and possible futures. NJ: Prentice Hall: Englewood Cliffs; 1986.

[75] Loersch C, Aarts H, Payne K, Jefferis V. The influence of social groups on goal contagion. J Exp Soc Psychol. 2008;44: 1555–1558. Available: http://www.sciencedirect.com/science/article/pii/S0022103108001170

[76] Shantz A, Latham G. An exploratory field experiment of the effect of subconscious and conscious goals on employee performance. Organ Behav Hum Decis Process. 2009;109: 9–17. Available: http://www.sciencedirect.com/science/article/pii/S0749597809000028

[77] Paivio A. Mental representations: A dual coding approach New York: Oxford University Press; 1990.

[78] Dodge K, Frame C. Gender and aggressive behavior. Child Dev. 1982;55: 163–173.

[79] Dodge K. Social cognition and children’s aggressive behavior. Child Dev. 1980;51: 162–170. 7363732

[80] Moran C. Purpose in Life, Student Development, and well-being: Recommandations for Student Affairs Practitioners. NASPA J. 2001;38: 269–280. Available: http://works.bepress.com/christy_moran_craft/5/

[81] Phillips DC, Orton R. The new causal principle of cognitive learning theory: Perspectives on Bandura’s”reciprocal determinism.” Psychol Rev. 1983;90: 158–165. Available: http://psycnet.apa.org/journals/rev/90/2/158/

[82] Jordan J, Mullen E, Murnighan JK. Striving for the moral self: the effects of recalling past moral actions on future moral behavior. Pers Soc Psychol Bull. 2011;37: 701–713. doi: 10.1177/0146167211400208 21402752 10.1177/0146167211400208

[83] Niemi RG, Hepburn MA, Chapman C. Community Service by High School Students: A cure for civic ills? Polit Behav. 2000;22: 45–69. doi: 10.1023/A:1006690417623

[84] Saha LJ. Prosocial Behaviour and Political Culture among Australian Secondary School Students. Int Educ J. 2004;5: 9–25. Available: http://eric.ed.gov/?id=EJ903832

[85] Stukas A. A., Snyder M., & Clary E. G. (1999). The effects of “Mandatory Volunteerism” on intentions to volunteer. Psychological Science, 10(1), 59–64.

[86] Gagné M. (2003). The Role of Autonomy Support and Autonomy Orientation in Prosocial Behavior Engagement. Motivation and Emotion, 27(3), 199–223.

[87] Deci E., & Ryan R. (2011). Self-determination theory In Van Lange P., Kruglanski A. W., & Higgins E. T. (Eds.), Handbook of theories of social psychology (pp. 416–437). Los Angeles: SAGE.

[88] Weinstein N., & Ryan R. M. (2010). When helping helps: autonomous motivation for prosocial behavior and its influence on well-being for the helper and recipient. Journal of Personality and Social Psychology, 98(2), 222–44. doi: 10.1037/a0016984 20085397 10.1037/a0016984

[89] Bandura A, Ross D, Ross S a. Imitation of film-mediated aggressive models. J Abnorm Soc Psychol. 1963;66: 3–11. doi: 10.1037/h0048687 13966304 10.1037/h0048687

[90] Gino F, Ayal S, Ariely D. Contagion and differentiation in unethical behavior: the effect of one bad apple on the barrel. Psychol Sci. 2009;20: 393–398. doi: 10.1111/j.1467-9280.2009.02306.x 19254236 10.1111/j.1467-9280.2009.02306.x

[91] Walton GM, Cohen GL, Cwir D, Spencer SJ. Mere belonging: the power of social connections. J Pers Soc Psychol. 2012;102: 513–32. doi: 10.1037/a0025731 22023711 10.1037/a0025731

[92] Aknin LB, Barrington-Leigh CP, Dunn EW, Helliwell JF, Burns J, Biswas-Diener R, et al Prosocial Spending and Well-Being: Cross-Cultural Evidence for a Psychological Universal. J Pers Soc Psychol. 2013;104: 635–652. doi: 10.1037/a0031578 23421360 10.1037/a0031578

[93] Mellor D, Hayashi Y, Firth L, Stokes M, Chambers S, Cummins R. Volunteering and Well-Being: Do Self-Esteem, Optimism, and Perceived Control Mediate the Relationship? J Soc Serv Res. 2008;34: 61–70. doi: 10.1080/01488370802162483

