# Supplementary material for: The influence of anonymous peers on prosocial behavior
Source: PLoS One. 2017 Oct 9;12(10):e0185521. doi: 10.1371/journal.pone.0185521 (PMC5633145; doi:10.1371/journal.pone.0185521)
Supplement: S1 Table — (DOCX) [file pone.0185521.s001.docx]

**S1 Table. Frequency of participants’ majors.**

| **College and Department** | | | | ***n* (%)** | |
| --- | --- | --- | --- | --- | --- |
| College of Social Sciences | | | | 24 (21.6%) | |
|  | Economics  Political Science and International Relations  Communication  Anthropology  Geography | | 13  6  3  1  1 | | |
| College of Engineering | |  | | 18 (14.4%) | |
|  | Mechanical and Aerospace Engineering  Architecture Engineering  Materials Science and Engineering  Chemical and Biological Engineering  Computer Science and Engineering  Electrical and Computer Engineering | | 6  3  3  3  2  1 | | |
| College of Humanities | | | | 17 (13.6%) | |
|  | English Language and Literature  Hispanic Language and Literature  Linguistic  Western History  Archaeology and Art History  Korean Language and Literature  Chinese Language and Literature  Russian Language and Literature  Korean History  Aesthetics | | 3  2  2  2  2  1  1  1  1  1 | | |
|  | Missing (i.e., answered as “Humanities”) | | 1 | | |
| College of Agriculture and Life Sciences | | | | 16 (12.8%) | |
| Food and Animal Biotechnology  Landscape Architecture and Rural System Engineering  Plant Science  Agriculture economics and Rural Development  Applied Biology and Chemistry | | | 6  4  3  2  1 | | |
| College of Education | | | | 14 (11.2%) | |
|  | English Education  Education  Social Studies Education  Geography Education  French Language Education  History Education  Earth Science Education  Ethics Education  Physical Education | | 3  2  2  2  1  1  1  1  1 | | |
| College of Business Administration | | | | 14 (11.2%) | |
| College of Natural Sciences | | | | 5 (4.0%) | |
|  | Physics and Astronomy  Mathematical Sciences  Statistics  Chemistry | | 2  1  1  1 | | |
| College of Nursing | |  | | 5 (4.0%) | |
| College of Human Ecology | | | | 4 (3.2%) | |
| Consumer and Child Studies  Food and Nutrition | | | 2  2 | | |
| College of Pharmacy | |  | | 4 (3.2%) | |
| College of Music | |  | | | 2 (1.6%) |
| Composition  Korean Music | |  | 1  1 | | |
| College of Medicine | |  | | 1 (0.8%) | |
| College of Fine Arts / Craft and Design | | | | 1 (0.8%) | |
| **Total** | |  | | **125 (100%)** | |
