# Supplementary material for: The influence of anonymous peers on prosocial behavior
Source: PLoS One. 2017 Oct 9;12(10):e0185521. doi: 10.1371/journal.pone.0185521 (PMC5633145; doi:10.1371/journal.pone.0185521)
Supplement: S1 Fig — (DOCX) [file pone.0185521.s002.docx]

**S1 Fig. Paragraphs excluding the presence of indirect peer influence**

| 1. My School Uniform  I took out my school uniform from the closet and sold it to a thrift store. I came up with this idea after thinking of what to do with my old uniform. I tried it on for the first time in ages and took pictures with a few friends of mine. It was real fun, just like the old days. We looked for ways to sell it and thought about which store would be best, hoping that everything would go all right. It was a great experience, and I even made some money!  2. Traveling Alone in Di Vero  It is the first time I am writing here :) I went on a solo trip to Di Vero last weekend to enjoy my first break from college to the fullest. It was a small town in the countryside that not many people had visited, so I felt nervous at first. But it turned out great! I learned a lot and felt really rewarded. Though I have just started traveling alone, I was so happy preparing for the trip and traveling around. I am up for even more adventures!  3. Cooking Class for College Students  Hi. I enjoy cooking in my spare time, so I recently signed up for a cooking class for college students. We take turns in looking for places to meet up and show off our cooking skills. It was my turn yesterday, and I made fried rice and sandwiches. I am having so much fun learning to make secret sauces and improving my own recipe these days. I haven't shared the new recipe with anyone yet! I thank those who organized this wonderful cooking class and others as well. I had such a great time. |
| --- |
