# Supplementary material for: The influence of anonymous peers on prosocial behavior
Source: PLoS One. 2017 Oct 9;12(10):e0185521. doi: 10.1371/journal.pone.0185521 (PMC5633145; doi:10.1371/journal.pone.0185521)
Supplement: S2 Fig — (DOCX) [file pone.0185521.s003.docx]

**S2 Fig. Paragraphs including the presence of indirect peer influence**

| 1. A Small Act of Kindness for Teenagers in Our Society  We are a group of college students interested in contributing to society. We think about ways to create a society where we all live in harmony and put these ideas into practice. This time, we came up with an idea to provide teenagers with custom-made uniforms. We looked for ways to get sponsors, hoping to make uniforms for teenagers who couldn’t afford to buy them. While trying to help others, we supported each other and became close friends as well.  2. A Visit to the Community Children's Center in Di Vero  It is the first time I am writing here :) I did volunteer work at the community children's center in Di Vero every weekend last month to make the most out of my first break from college. I was worried about whether I could do a good job at first, but the children were so nice and kind. I learned a lot from them and felt really rewarded. Though I have just started volunteering, I am so happy helping others.  3. Sharing Good Food with Good People  Hi. I enjoy cooking in my spare time, so I recently signed up for a volunteer group made up of college students to support the underprivileged. And yesterday, I visited the local children's welfare center and made some good food for children in need. I made fried rice and sandwiches, hoping to be of help at least a little bit. I thank the leader of our volunteer group and other members as well. The children and all the people I met were really nice, so I had a great time. |
| --- |
