# Supplementary figures and images for: The influence of anonymous peers on prosocial behavior
Source: PLoS One. 2017 Oct 9;12(10):e0185521. doi: 10.1371/journal.pone.0185521 (PMC5633145; doi:10.1371/journal.pone.0185521)

**S3 Fig. Setup of laboratory experiment**

**
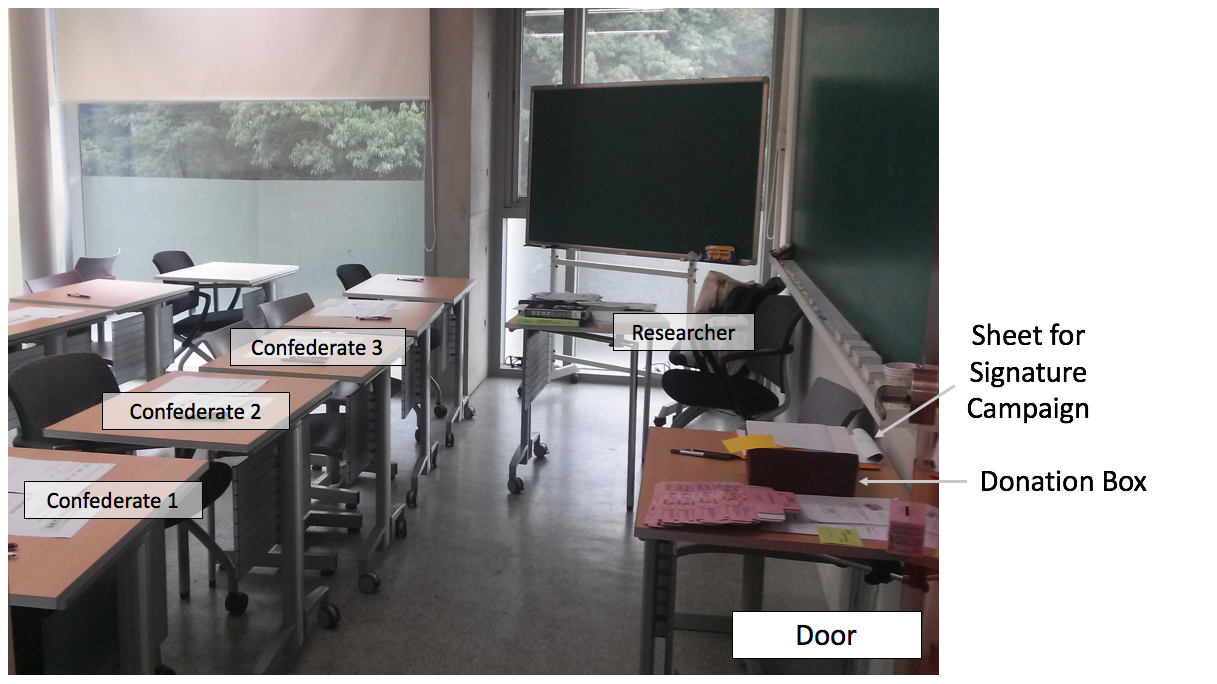
**

Supplement: S3 Fig — (DOCX) [file pone.0185521.s004.docx]
