# Supplementary material for: The influence of anonymous peers on prosocial behavior
Source: PLoS One. 2017 Oct 9;12(10):e0185521. doi: 10.1371/journal.pone.0185521 (PMC5633145; doi:10.1371/journal.pone.0185521)
Supplement: S4 Fig — (DOCX) [file pone.0185521.s005.docx]

**S4 Fig. Paragraph requesting prosocial behavior**

| Okay, that's all for today. Thank you for participating in the study. Oh, before you leave, there is one more thing I would like to say. As a clinical researcher at a hospital, I often come across sick babies and children. I feel so sad looking at babies not getting better just because they don't have money to get treatment. Of course it's important to concentrate on my studies, but I thought I should look around and do what I can do. So I started to sponsor a child, and now I'm asking SNU students for donations and signatures to support the increase of funding for child patients. (point at the sheets)  It's 100% up to you to make a decision. I'm not forcing you, so please don't feel obligated or anything. If you do wish to contribute, 1,000-2,000 won or even just 100-200 won would be appreciated. Here's the signature sheet (point again), so please join us if you want to! Thank you. |
| --- |
